# Complement C5a Implication in Axonal Growth After Injury

**DOI:** 10.3390/cells13201729

**Published:** 2024-10-18

**Authors:** Aurélie Cotten, Charlotte Jeanneau, Patrick Decherchi, Imad About

**Affiliations:** Aix-Marseille University, CNRS, ISM, 13009 Marseille, France; aurelie.cotten@univ-amu.fr (A.C.); charlotte.jeanneau@univ-amu.fr (C.J.); patrick.decherchi@univ-amu.fr (P.D.)

**Keywords:** Complement C5a, neuron injury, axotomy, axon regeneration

## Abstract

Complement C5a protein has been shown to play a major role in tissue regeneration through interaction with its receptor (C5aR) on target cells. Expression of this receptor has been reported in the nervous system which, upon injury, has no treatment to restore the lost functions. This work aimed at investigating the Complement C5a effect on axonal growth after axotomy in vitro. Primary hippocampal neurons were isolated from embryonic Wistar rats. Cell expression of C5aR mRNA was verified by RT-PCR while its membrane expression, localization, and phosphorylation were investigated by immunofluorescence. Then, the effects of C5a on injured axonal growth were investigated using a 3D-printed microfluidic device. Immunofluorescence demonstrated that the primary cultures contained only mature neurons (93%) and astrocytes (7%), but no oligodendrocytes or immature neurons. Immunofluorescence revealed a co-localization of NF-L and C5aR only in the mature neurons where C5a induced the phosphorylation of its receptor. C5a application on injured axons in the microfluidic devices significantly increased both the axonal growth speed and length. Our findings highlight a new role of C5a in regeneration demonstrating an enhancement of axonal growth after axotomy. This may provide a future therapeutic tool in the treatment of central nervous system injury.

## 1. Introduction

Axonal growth has been well investigated during neurodevelopment, where multiple attractive/permissive as well as repulsive/inhibitory signals guide axon pathfinding and target recognition. Decisions to continue a particular path, turn, collapse, or retract depend on receptors on the growth cones, located at the axon’s extremity. These include cell adhesion molecules [1], repulsive molecules [2], and soluble molecules, which act as guidance signals directing the axonal growth [2]. While several guidance factors and their receptors persist in adulthood, the axonal regeneration potential in the mature neurons is limited [3,4]. Additionally, upon injury of the adult central nervous system (CNS), it is difficult to achieve/enhance axonal regeneration. Strategies have been developed to enhance axonal regeneration after CNS injury. These were mainly based on using the residual and intact neuronal networks to stimulate and guide axonal growth over long distances in an inhibitory environment to form functional synapses with the appropriate targets [5]. Among these strategies, pharmacological treatments [6,7] combined with tissue grafting [8,9] and scaffolds [10,11] have been developed. However, to date, there is still no effective pharmacological strategy to restore the lost functions. Recently, attention has turned to target the Complement system.

The Complement system is made of 35 proteins mainly synthesized by the liver [12]. Its functions are well demonstrated during the inflammation process. Indeed, infection and tissue injury are known to activate the Complement system, leading to the production of active fragments. Among these, C3a and C5a act as anaphylatoxins. They increase vessel permeability and allow the recruitment of inflammatory cells expressing their receptors to eliminate cell debris and pathogens [13]. Another fragment, C3b, opsonizes the pathogens and enhances their phagocytosis by the recruited and resident phagocytes [14], while the membrane attack complex (C5-b9), which is made of several proteins, polymerizes in the bacterial wall creating tunnel-shaped structures. These allow loss of electrolytes and the massive entry of extracellular fluids, leading to the pathogen lysis [15]. Since the Complement system initiates the inflammatory reaction, which hinders the axonal regeneration, Complement protein C5a was targeted in several studies with the idea that inhibiting the inflammation would allow axonal regeneration [16,17].

Interestingly, the expression of Complement receptors on cell types other than the inflammatory cells has attracted much attention. This led to exploring the Complement implication in other functions, and especially in the regeneration process [12,18]. Indeed, previous studies provided evidence supporting the chemotactic role of C5a through interaction with its G-protein-coupled receptor (C5aR) in the regeneration of the liver, muscles, bones, heart, and skin [12]. C5aR expression has also been reported in the central nervous system (CNS). During development, its expression has been demonstrated in mouse embryonic neural progenitor cells in vivo and on human embryonic stem cell-derived neural progenitors [19]. In situ hybridization studies coupled with immunohistochemical approaches revealed that most neurons in the hippocampal formation, many pyramidal cortical neurons, and cerebellar Purkinje neurons in normal human and murine brains constitutively express C5a receptors [17,20,21,22]. Thus, while C5a is involved in the inflammatory process, its expression in the CNS suggests its implication in other processes such as development and regeneration.

Indeed, depending on the concentrations used, C5a has been shown to induce neurite growth of intact mice neurons in vitro [23]. More recently, upon stimulation with lipoteichoic acid, human pulp fibroblasts were found to produce C5a. The interaction of this molecule with the C5aR of these cells mediated the local brain-derived neurotrophic factor (BDNF) secretion, which guided the neuronal growth toward the lipoteichoic acid-stimulated fibroblasts [24].

We, therefore, hypothesized that C5a may promote the axonal growth of damaged neurons. Based on this hypothesis, we investigated the neuronal expression of C5aR on hippocampal neurons, and the effect of C5a on its phosphorylation and on the growth of axotomized neurons, by measuring the axonal growth length and speed in vitro.

## 2. Materials and Methods

### 2.1. Animals

Hippocampal cells were isolated from embryonic Wistar rats (E18.5) and spinal cords from pregnant Wistar rats (*n* = 30), weighing 250 to 300 g (Centre d’Élevage Roger JANVIER^®^, Le Genest Saint Isle, France). A veterinarian performed animal sacrifices without any injected substances that could affect the cell cultures.

### 2.2. Experimental Design

**Figure 1:**
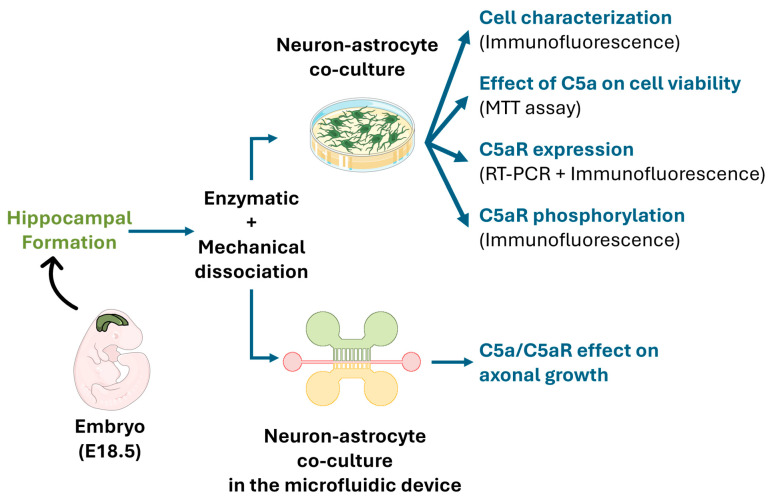
A schematic view of the experimental design. Cells were obtained from the hippocampal formation of rat embryos (E18.5) using enzymatic and mechanical dissociation methods. Neuron-astrocyte co-cultures were used to investigate C5aR expression/activation and the effect of C5a on cell viability. The microfluidic device was used to quantify the C5a effect on axonal growth.

In the first step, we cultured rat E18.5 embryonic hippocampal neurons, obtained by enzymatic and mechanical dissociation. Then, cell characterization, and C5aR expression and activation, were determined by immunostaining. RT-PCR and immunostaining were used to determine the C5aR mRNA and protein expression. C5a toxicity was evaluated by MTT assay. Then, a microfluidic device was fabricated using soft lithography, and the injured axonal growth speed and length were measured under the expression of a Green Fluorescent protein (GFP) using ImageJ and a tracing plugin after applying a C5a gradient (Figure 1).

### 2.3. Reagents and Materials

Cell culture media, supplements, laminin, and immunofluorescence mounting medium were purchased from ThermoFisher Scientific (Illkirch-Graffenstaden, France) while cell culture materials were from Dominique Dutscher (Bernolsheim, France). Rabbit IgG against phosphorylated rat C5aR (AY-AF8362) was purchased from Euromedex (Souffelweyersheim, France); rabbit IgG anti-rat C5aR (CPA4586) from CliniSciences (Nanterre, France); Alexa Fluor 594 goat anti-rabbit IgG (A11012), Alexa Fluor 488 goat anti-rabbit IgG (A11034), Alexa Fluor 594 donkey anti-mouse IgG (A21203), and rabbit IgG anti-rat NF-L (PA587394) from ThermoFischer Scientific; mouse IgG anti-rat glial Fibrillary Acidic Protein (MAB360) from Merck Millipore (Darmstadt, Germany); and mouse IgG anti-rat oligodendrocyte transcription factor (sc-515947) and mouse IgG anti-rat neuronal Differentiation 2 (sc-365896) from Santa Cruz Biotechnology (Heidelberg, Germany). Poly-D-lysin was purchased from Sigma Aldrich (St. Louis, MO, USA).

### 2.4. Cell Cultures and Characterization

#### 2.4.1. Primary Neuron Culture

Culture supports were coated with Poly-D-lysin (0.1 mg/mL) and laminin mouse protein (10 µg/mL) overnight at 4 °C. Hippocampal structures were isolated at 4 °C, washed 2 times in fresh HBSS/10 mM HEPES solution, and incubated at 37 °C for 15 min in HBSS/10 mM HEPES 0.25% trypsin for enzymatic cell dissociation. Mechanical dissociation was then performed with two different dimensions of a sterile pastor pipette precoated with fetal bovine serum (FBS). The supernatant was centrifuged for 5 min at 0.1 relative centrifugal force (rcf) and the cellular pellet was suspended in 1 mL of Neurobasal^TM^ medium 2% B-27^TM^ supplemented with 1% Penicillin-Streptomycin (100 UI/mL) and 1% L-glutamine (0.5 mM) preheated at 37 °C. Cells were seeded in the same medium and incubated at 37 °C, 5% CO_2_, and 95% humidity.

#### 2.4.2. Cell Characterization

Cells were cultured (1.5 × 10^4^ cells/cm^2^) on glass coverslips. Then, after 14 days of culture, they were characterized by immunostaining. The cells were fixed for 20 min in 4% paraformaldehyde (PFA) containing 4% sucrose prewarmed at 37 °C. After two washes with phosphate buffer (PBS), cells were permeabilized and nonspecific binding sites were blocked for 1 h at room temperature with 0.1 M PBS (pH 7.4), 0.22% gelatin, and 0.1% Triton X-100. The primary antibodies used were mouse IgG anti-rat glial fibrillary acidic protein (GFAP, 0.4 µg/mL), rabbit IgG anti-rat NF-L (7.9 µg/mL), mouse IgG anti-rat oligodendrocyte transcription factor (OLIG2, 0.4 µg/mL), and mouse IgG anti-rat neuronal Differentiation 2 (NeuroD2, 0.4 µg/mL). The primary antibodies and their respective control IgG were incubated overnight at 4 °C. The cells were then incubated with secondary antibodies: Alexa Fluor 488 goat anti-rabbit IgG and donkey anti-mouse IgG, and Alexa Fluor 594 goat anti-rabbit IgG, while nuclei were labeled with 4′,6-diamidino-2-phenylindole (DAPI, 1 µg/mL) for 1 h at room temperature. Images were acquired with an epifluorescence microscope (Axio Observer Z1, Carl Zeiss S.A.S., Marly Le Roi, France). The labeled cells were counted in three different fields at 20×, and the results are expressed as a percentage of the total cell count.

### 2.5. C5aR Expression

#### 2.5.1. Reverse Transcriptase Polymerase Chain Reaction (RT-PCR)

Neurons were cultured in 6-well plates (4.5 × 10^4^ cells/cm^2^). After 14 days, the cells were injured with a sterile scalpel by performing 5 parallel horizontal and 5 perpendicular lines. Total RNA from injured and intact neurons was isolated using a PureLink RNA mini kit (Life Technologies, Oslo, Norway) according to the manufacturer’s indications. Purified RNA was reverse transcribed into cDNA using the reverse transcription system kit (Promega, Madison, WI, USA). The primer sequences used are listed in Table 1. PCR products were separated onto 1% agarose gel using E-Gel^TM^ Imager Blue-Light Base (ThermoFisher Scientific). Data were analyzed using a GelCapture image Analysis software version 4.25 (ThermoFischer Scientific). GAPDH gene expression was used as the internal control. 

#### 2.5.2. Spinal Cord Isolation and Histological Processing

The cervical spinal cord was extracted from Wistar rats, routinely processed, and cryosectioned after discarding the meninges (*n* = 3). Immediately after rat sacrifice, the spinal cord was extracted with the meninges (C2-T12), fixed in 4% PFA overnight at room temperature, and conserved for 1 week in fresh 4% PFA at 4 °C. Then, tissular segments were removed from the meninges and cryoprotected with 0.1 M PBS 30% sucrose for 5 days at 4 °C before being frozen at −80 °C. Cryosection was performed with cryostat at a 5 µm thickness. Cervical sections were recovered on slides doubly gelatinized with distilled H_2_O 0.1% porcine gelatin 0.5% Chromium (III) potassium sulfate dodecahydrate (Crk(SO_4_)_2_).

#### 2.5.3. Immunostaining

Double immunostaining was performed on intact and injured neurons (1.5 × 10^4^ cells/cm^2^) cultured on a glass coverslip for 14 days. First, rabbit IgG anti-rat NF-L and Alexa Fluor 594 goat anti-rabbit IgG (1 µg/mL) antibodies were used, and rabbit IgG anti-rat C5aR (0.7 µg/mL) and Alexa Fluor 488 goat anti-rabbit IgG (1 µg/mL) antibodies were applied with DAPI (1 µg/mL). Controls were prepared by incubating the cells with unrelated IgG isotypes.

Similarly, double immunostaining was performed on spinal cord cryosection to localize the NF-L in red and C5aR in green. Spinal cord sections were permeabilized and non-specific binding sites were blocked overnight with 0.1 M PBS (pH 7.4) 0.22% gelatin, and 0.1% Triton X-100. Sections were then incubated with primary antibodies of rabbit anti-rat NF-L, (1.18 µg/mL), rabbit IgG anti-rat C5aR (1.34 µg/mL), or control IgG for 48 h at 4 °C in a humidity chamber. Controls were performed by omitting the primary antibodies and nuclei were counterstained in blue using DAPI. After 3 washes (2 h) in 0.1 M PBS (pH 7.4) 0.22% gelatin, and 0.1% Triton X-100 at 30 rpm, sections were incubated for 1 h at room temperature with the secondary antibodies Alexa Fluor 488 goat anti-rabbit IgG and 594 goat anti-rabbit IgG (0.25 µg/mL), and with DAPI (1 µg/mL). Spinal cord sections were washed 3 times for 2 h before being mounted with ProLong Glass^TM^ (ThermoFischer Scientific).

### 2.6. C5a and Cell Viability 

Neurons (3.5 × 10^4^ cells/cm^2^) cultured in 48-well plates for 14 days were injured (as described above). After 30 min, cells were incubated for 24 h at 37 °C in a medium containing 3 concentrations of C5a (50, 200, and 1000 ng/mL). After 24 h and 48 h, an MTT assay was performed by incubating cells with (3-(4,5-dimethylthiazol-2-yl)-2,5-diphenyltetrazolium bromide (50 ng/mL) for 2 h at 37 °C. The supernatants were discarded and 350 µL of dimethyl sulfoxide (DMSO, 100%) was added to each well. The optical density at 550 nm was recorded using a spectrometer (Σ960, Metertech, Taipei, Taiwan) and normalized to the number of surviving cells obtained at 2 h incubated in medium without C5a.

### 2.7. C5aR Phosphorylation

Neurons (6.5 × 10^4^ cells/cm^2^) were seeded in 8-well culture plates (Lab-Tek^TM^, ThermoFisher Scientific, Illkirch-Graffenstaden, France). After 14 days, the cells were incubated in the culture medium for 1 h at 37 °C with the PMX53 (R&D Systems, Lille, France) at 500 ng/mL to ensure the inhibition of C5a fixation in conditions with the PMX53. Then, the cells were incubated for 5 min with the culture medium ± C5a (50 ng/mL) or with the C5a and PMX53 inhibitor. The cells were fixed with 4% PFA containing 4% sucrose for 20 min, blocked with PBS 4% BSA for 1 h, and permeabilized with PBS 0.1% Triton X-100 for 2 h at room temperature. Finally, immunostaining was performed using rabbit IgG anti-rat C5aR phosphorylated and rabbit IgG anti-rat NF-L as the primary antibodies, and Alexa Fluor 594 goat anti-rabbit IgG and goat anti-rabbit IgG 488 as the secondary antibodies.

### 2.8. Axonal Regeneration

#### 2.8.1. Microfluidic Device Conception and Fabrication

**Figure 2:**
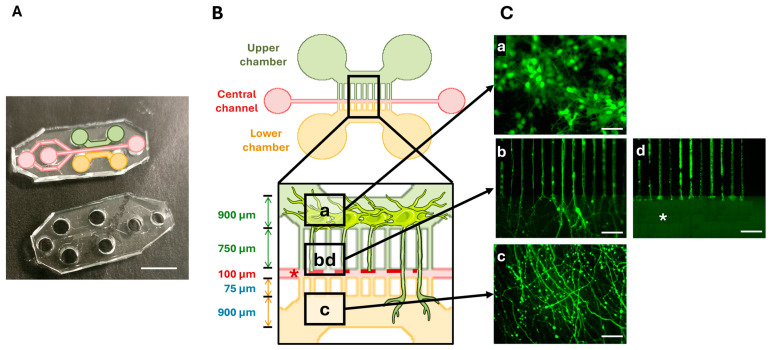
The microfluidic device. (**A**) A photography of the Polydimethylsiloxane (PDMS)-printed cell culture device. (**B**) A schematic drawing of the microfluidic device. The upper (green) and lower (orange) chambers are connected to a central channel (red) via 3 µm wide microchannels. Boxes indicate the regions of interest. (**C**) Cell culture and injury in the microfluidic device. The upper chamber contained green GFP-labeled neurons with cellular bodies measuring around 15 µm in diameter (a). The axons extend through the microchannels to the lower compartment (b). The lower chamber contained neurites (c). Injury to the axons was performed in the central channel (* and d). Scale bars: (**A**): 5 mm; (**C**): 50 µm.

The microfluidic device was developed by the Institut de Neurophysiophatologie (Timone, Marseille, France). It was fabricated using soft lithography as previously described [25,26]. First, a master mold of the chamber and microchannel was fabricated in polymerized resin (type R123, Soloplast, Vosschemie, France). Then the Epoxy molds were filled with Polydimethylsiloxane (PDMS) silicone (SYLGARDTM 184 Silicone Elastomer kit, Dow Silicones, Germany) and polymerized at 70 °C for 90 min. The silicon-containing chamber and microchannel stamps were de-molded and cleaned in ethanol with ultrasound for 1 min at 40 kHz (Ultrasonic Cleaner, BPAC, Vern-sur-Seiche, France). The cut PDMS and Petri dishes glass-bottom (FluoroDish, WPI) were placed into plasma cleaner (Diener Electronic, Ebhausen, Germany) under vacuum for 60 s for surface activation. They were brought together to form a tight seal by incubating them for 30 min in an oven at 70 °C as described [26]. Then, they were plasma cleaned a second time for 6 s. This device was developed to culture neurons in the upper compartment and to allow axonal growth through 3 µm diameter microchannels to the lower compartment. The device also comprises a central channel designed perpendicular to the microchannels to perform the axotomy (Figure 2).

#### 2.8.2. Cell Culture in the Microfluidic Device

The device surface was treated as previously explained for cell cultures and the cells were seeded at 140 cells/cm^2^ in the upper chamber for 3 h. The neurons were then infected with GFP lentivirus [26] 3 h after seeding and incubated for 14 days at 37 °C. Then, a mechanical axotomy was performed in the central channel on the mature axons by vacuum aspiration. The process was repeated 5 times to ensure a complete axotomy. After 30 min of incubation, a medium ± PMX53 (500 ng/mL) was added to the two compartments for 1 h. Subsequently, the compartments were emptied, and 40 µL of medium with C5a (50 ng/mL) or PMX53 and C5a was added to the lower chamber and 20 µL of medium without C5a into the upper one. A differential pressure was created by a volume difference, establishing a gradient between the two chambers 2 h after adding the treatment. The treatment conditions were medium (control), C5a (50 ng/mL), and C5a (50 ng/mL) + PMX53 (500 ng/mL). All these experiments were performed 12 times to decrease inter- and intra-variability. Live-cell imaging was performed using a microscope and a fluorescence filter at 450–490 nm (40×/1.3 Oil DICII, Optovar 1×) connected to a camera using MetaMorph software version 7.10.2.240 64-bit, (Molecular Devices, San Jose, CA, USA). The region of interest (ROI) includes the central canal and the axonal compartment. ROI pictures at multiple steps were taken in these compartments in each device (20 × 3 pictures). These steps were performed at 0 h, 24 h, and 48 h after the gradient establishment.

#### 2.8.3. Image Analysis

The ROI was reconstructed by creating a mosaic of all pictures in one chip using the MosaicJ plugin in ImageJ software 1.53k (Java 1.8.0_172 64-bit, National Institutes of Health, Bethesda, MD, USA). The cumulative length of axonal growth (*n* = 200 axons/conditions) was measured as a function of time by image analysis using the NeuronJ 1.4.3 plugin [27]. Values were collected in Excel version 2409 (Microsoft Ireland Operations Ltd., Dublin, Ireland) and the median replaced the values outside the interquartile range. The speed was calculated by the ratio of the axon length difference by the time (µm/h). All data were normalized as a percent of the control condition (Medium at 0 h).

### 2.9. Statistical Analysis

All experiments were performed at least in triplicate. Statistical analysis was performed using RStudio 1.4.1106 (Rasband, W.S., U. S. National Institutes of Health, Bethesda, MD, USA). After the Shapiro test, Friedman tests were conducted to assess temporal changes, and Kruskal–Wallis tests for condition comparisons with Bonferroni correction for multiple comparisons to study axonal growth speed and length. Additionally, Wilcoxon post hoc tests were performed to examine specific differences between conditions. Data are expressed as means ± SD and were considered significant for *p*-value < 0.05. Cell percentages were analyzed by performing the Shapiro, Kruskal–Wallis, and Wilcoxon post hoc tests with Bonferroni correction for cell culture characterization.

## 3. Results

### 3.1. Cell Culture Mainly Contained Neurons

**Figure 3:**
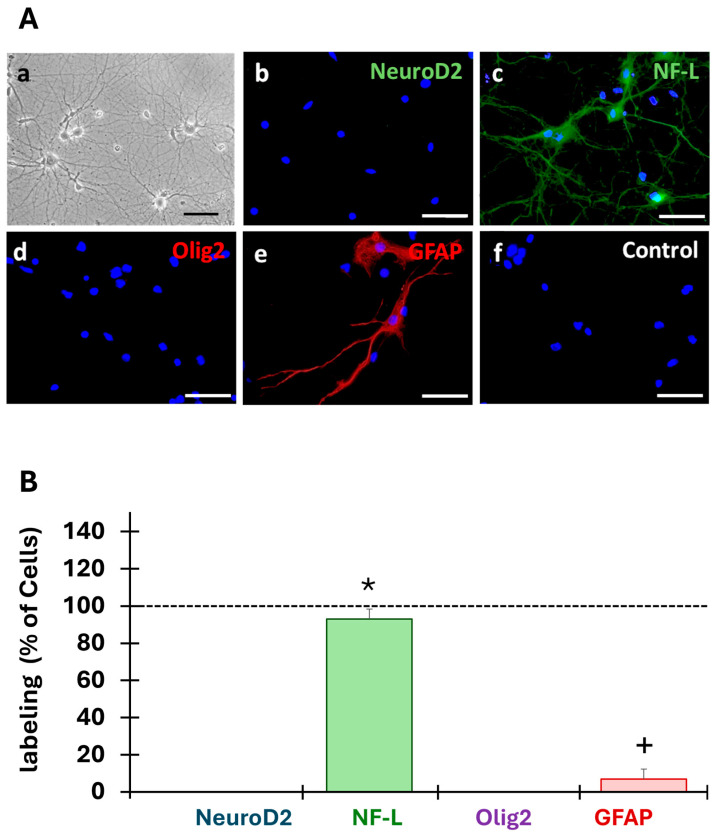
Characterization of the primary cell cultures. (**A**) After 14 days of culture, the cells obtained from embryonic hippocampal rat formations (*n* = 3) developed extensive neurites (**a**). Expression of markers of undifferentiated (NeuroD2) and differentiated (NF-L) neurons, astrocytes (GFAP), and oligodendrocytes (Olig2) was investigated by immunofluorescence. NF-L (**c**) and GFAP (**e**) were expressed in the primary cultures but not NeuroD2 (**b**) nor Olig2 (**f**). No staining was observed in the control condition (**d**). Scale bars: 50 µm. (**B**) The cells labeled with the different markers were counted in three fields (20×) and were expressed as a percentage of all cells (DAPI, dotted lines at 100%). * Indicates a significant (*p* < 0.05) difference between the percent of NF-labeled cells and the other conditions, and + indicates a significant (*p* < 0.05) difference between the percentage of GFAP-labeled cells with other conditions.

The cells obtained from embryonic hippocampal rat formations (*n* = 3) developed extensive neurites (Figure 3A(a)). Immunofluorescence staining revealed that the cultured cells expressed the neuron intermediated filaments marker NF-L (Figure 3A(c)), specific to differentiated neurons, but did not express NeuroD2, the undifferentiated neuron marker (Figure 3A(b)). Furthermore, data analysis indicated that cells did not express the oligodendrocyte marker (oligodendrocyte transcription factor, Olig2) (Figure 3A(d)) but expressed the glial fibrillary acidic protein (GFAP) astrocyte marker (Figure 3A(e)). No staining was detected in the controls (Figure 3A(f)).

Analysis of the obtained cell populations revealed that our primary cultures were composed of 93 ± 5% of differentiated neurons (NF-L) and 7 ± 5% of astrocytes (GFAP) (Figure 3B). Kruskal–Wallis (H(3) = 47.09, *p* = 3.331 × 10^−10^), and Wilcoxon’s post hoc test revealed that the primary culture was a mixed population of cells mainly composed of differentiated neurons (93%), astrocytes (7%), and other cell populations.

### 3.2. Neurons Expressed the C5aR

**Figure 4:**
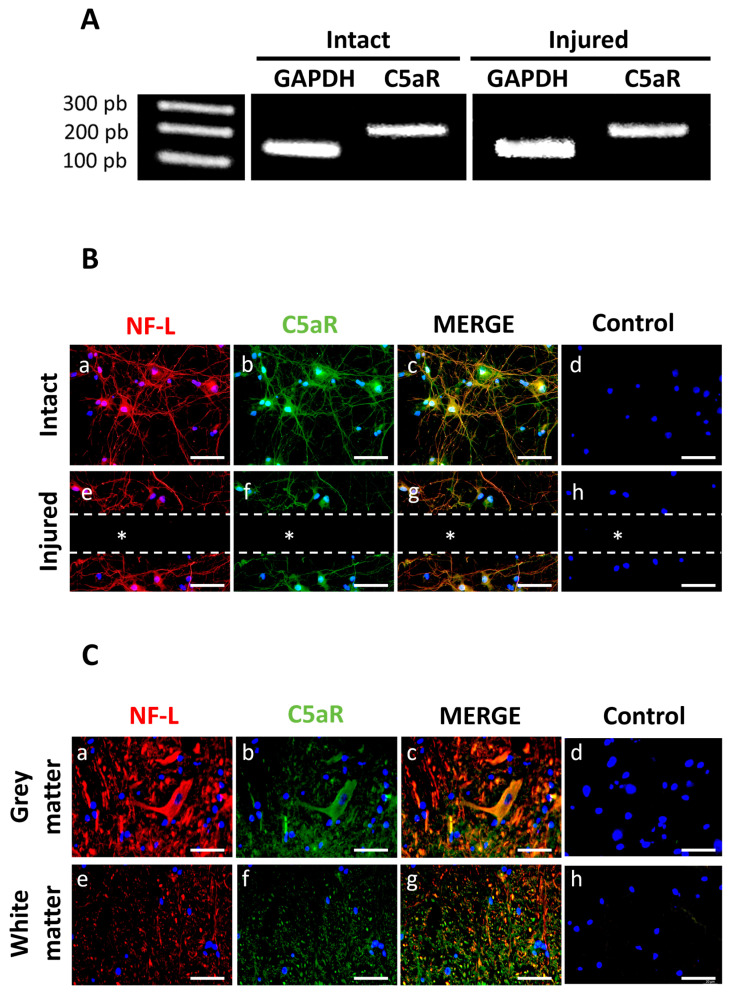
C5aR expression in neuron-astrocyte co-cultures by RT-PCR. (**A**) C5aR mRNA expression was observed in both injured and intact cell cultures. Controls included expression of housekeeping GAPDH mRNA. (**B**) Double immunostaining for NF-L (green) and C5aR (red) indicated that injured and intact neurons expressed NF-L (**a**,**e**) and C5aR (**b**,**f**) on their membranes (*n* = 3). Merged images in yellow (**c**,**g**) revealed a co-localization of these markers. Controls for each condition were performed by omitting the primary antibodies (**d**,**h**) and nuclei were counterstained using DAPI. The dotted lines and * indicate the site of injury. Scale bars: 50 µm. (**C**) Double immunostaining of NF-L in red (**a**,**e**) and C5aR in green (**b**,**f**) on spinal cord cryosections. Controls were performed by omitting the primary antibodies (**d**,**h**) and nuclei were counterstained in blue using DAPI. Images revealed that C5aR was co-expressed with NF-L in grey and white matters (yellow in (**c**,**g**)). Scale bars: 50 µm.

RT-PCR analysis on differentiated neuron cultures showed that C5aR mRNA was expressed in both injured and intact cultures (Figure 4A). Immunostaining revealed that the NF-L marker and C5aR were expressed and localized in the membrane around the cell bodies and cell extensions (Figure 4B(a,b)). The merged images indicated that these expressions were co-localized (Figure 4B(c)). No labeling was observed in the control condition (Figure 4B(d,h)) and the area on the lesion (Figure 4B(e–g)). In vivo, C5aR was strongly expressed in cells expressing NF-L in the grey and white matters (Figure 4C(a,b,e,f)). Merged images show a co-expression of C5aR and NF-L both in the grey and white matters (Figure 4C(c,g)), while no labeling was observed in the controls (Figure 4C(d,h)).

### 3.3. C5a Did Not Affect Cell Viability

**Figure 5:**
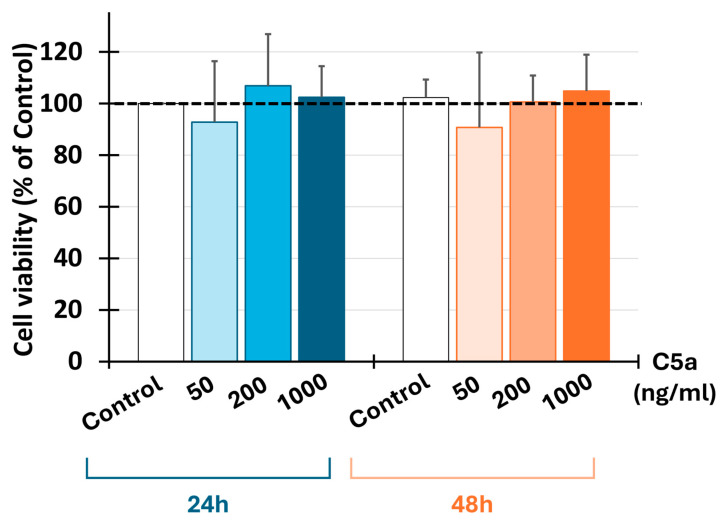
C5a and cell viability. Injured neural cell viability was determined by MTT assay after 24 and 48 h of culture (*n* = 5) using three C5a concentrations: 50 ng/mL, 200 ng/mL, and 1000 ng/mL. The results are expressed as a percentage of control (dashed line). C5a did not affect the cell viability after 24 or 48 h.

The use of C5a at all determined concentrations (50, 200, and 1000 ng/mL) did not affect the cell viability at all tested concentrations for 24 or 48 h (Figure 5).

### 3.4. C5a Induced C5aR Phosphorylation

**Figure 6:**
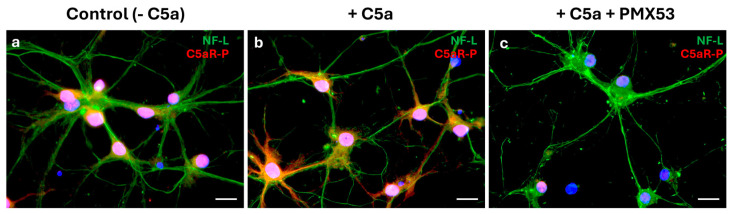
Interaction between C5a and neuron C5aR. Cells were cultured in the B-27-supplemented neurobasal medium as a control condition without C5a (**a**), stimulated with C5a (50 ng/mL) (**b**) or with both C5a and PMX53 (**c**) for 5 min (*n* = 3). Immunostaining of the phosphorylated C5a receptor (C5aR-P, red) and NF-L (green) indicated that while NF-L was expressed in all conditions, C5aR-P was not expressed by cells when the inhibitor was added. Scale bars: 20 µm.

Immunofluorescence demonstrated a co-localization of phosphorylated C5aR (C5aR-P) with the mature neuron NF-L marker (Figure 6a). Adding C5a to the differentiated cell cultures phosphorylated the C5aR-P on these cells (Figure 6b). When the C5aR inhibitor (PMX53) was added together with the C5a, immunofluorescence demonstrated an absence of phosphorylation of the C5a receptor (Figure 6c).

### 3.5. Culture of Neurons in the Microfluidic Device and Axonal Growth

Two days after cell seeding, GFP protein was added to the neurons in the microfluidic device (Figure 2C). The cell bodies, measuring around 15 µm, were maintained in the upper chamber (Figure 2C(a)), whereas the axons grew through the microchannels (Figure 2C(b)) into the lower chamber (Figure 2C(c)). The mechanical injury was performed in the central channel where no axons were detectable after the vacuum aspiration was performed (Figure 2C(b)).

### 3.6. C5a Increased the Axonal Growth

**Figure 7:**
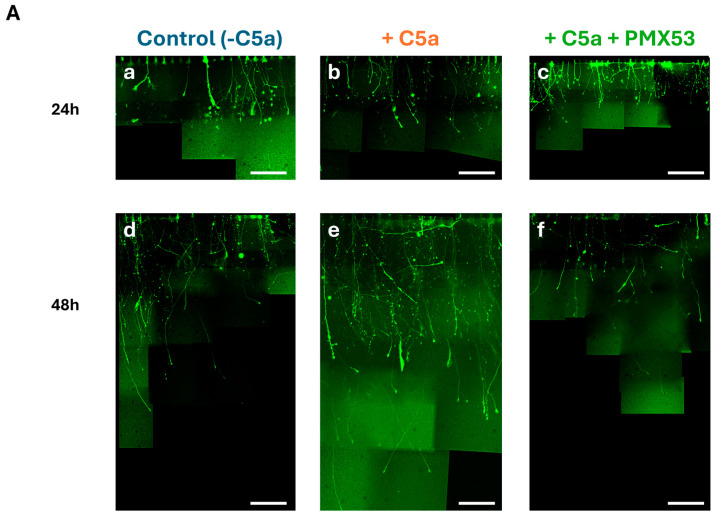

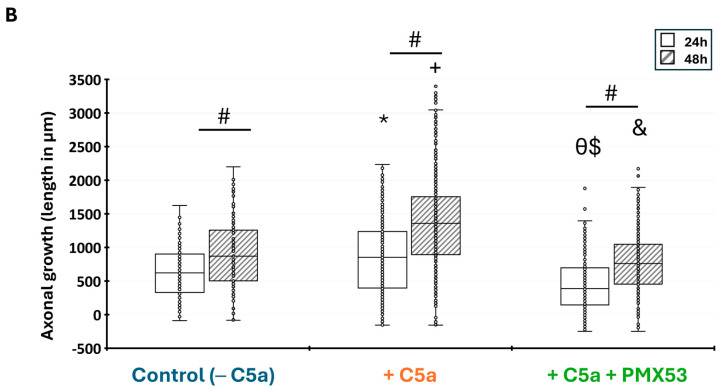
Axonal growth in the microfluidic device. (**A**) Cells were incubated with the control medium for 48 h either without C5a (**a**,**d**), or with C5a (50 ng/mL) (**b**,**e**), or with both C5a and PMX53 (**c**,**f**). GFP labeling allowed visualization of the axonal growth via pictures taken at 40×. The axonal growth is reported by combining the images obtained over the 48 h growth period. Scale bars: 100 µm. (**B**) After axonal injury, the axonal length significantly increased under all conditions between 24 h (white) and 48 h (hatching). Adding C5a significantly increased the axonal length at 24 h and at 48 h as compared to the controls at the same delays. This increase was suppressed after adding the C5aR inhibitor. Results are presented as boxplots. The central line in each boxplot represents the median values while the box edges indicate the first and third quartiles. All points beyond the whiskers up to 1.5 times the interquartile range are considered as outliers. # indicates a significant (*p* < 0.05) difference between incubation periods for the same treatment. * and + indicate significant (*p* < 0.05) differences between the axonal lengths after adding C5a as compared to the control at 24 and 48 h, respectively. $ and & indicate significant (*p* < 0.05) differences with C5a + PMX53 compared to the C5a treatment at 24 and 48 h, respectively. Ө indicates significant (*p* < 0.05) differences with C5a + PMX53 compared to the control at 24 h.

After axotomy, the axonal growth significantly increased (*p* < 0.05) between 24 (Figure 7A(a–c),B) and 48 h (Figure 7A(d–f),B) under all culture conditions. Kruskal–Wallis tests revealed significant differences between the conditions at both 24 h (H = 102.04, df = 2, *p* = 2.2 × 10^−16^) and 48 h (H = 134.02, df = 2, *p* = 2.2 × 10^−16^). The axonal growth with C5a was significantly higher than without C5a both after 24 h and 48 h (Figure 7B) (*p* < 0.05). This axonal growth increase was suppressed after the addition of the C5aR inhibitor (PMX53), leading to values comparable to those of the control at both periods (Figure 7A(c,f),B).

### 3.7. The Effect of C5a Treatment on Axonal Growth Speed

**Figure 8:**
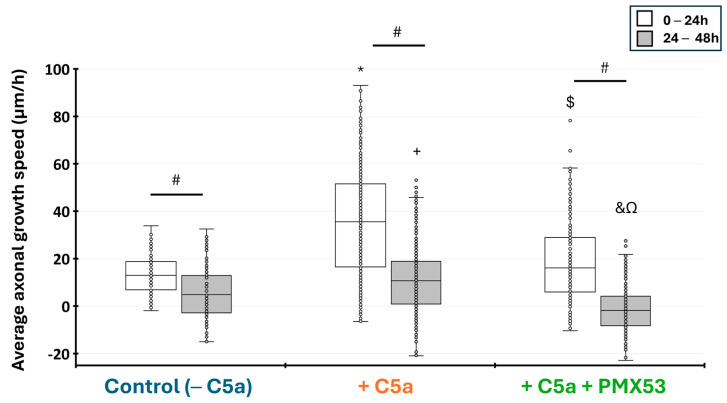
Injured axonal growth speed in the microfluidic device. The average growth speed (µm/h) was measured between 0–24 h and 24–48 h after the axonal injury. The axonal growth speed was higher during the first 24 h and slower from 24 to 48 h with all culture conditions. This speed significantly increased when C5a was added to the injured neurons for the periods from 0 to 24 h and from 24 to 48 h. Adding PMX53 and C5a simultaneously significantly decreased the axonal growth speed at both time periods. Results are presented as boxplots. The central line in each boxplot represents the median values while the box edges indicate the first and third quartiles. All points beyond the whiskers up to 1.5 times the interquartile range are considered as outliers. # indicates a significant (*p* < 0.05) difference between incubation periods for the same treatment. * and + indicate significant (*p* < 0.05) differences after between the axonal growth speed after adding C5a as compared to the control at 0–24 h and 24–48 h, respectively. $ and & indicate significant (*p* < 0.05) differences with C5a + PMX53 compared to the C5a treatment at 0–24 h and 24–48 h, respectively. Ω indicates significant (*p* < 0.05) differences with C5a + PMX53 compared to the control at 24–48 h.

Measurement of the axonal growth showed that C5a significantly enhanced the axonal growth speed from 0–24 h and 24–48 h hours as compared to the control without C5a (*p* < 0.05). The Kruskal–Wallis test revealed highly significant differences between conditions at 0–24 h (H = 147.38, df = 2, *p* = 2.2 × 10^−16^) and 24–48 h (H = 121.46, df = 2, *p* = 2.2 × 10^−16^). This speed significantly decreased between 24 and 48h with the control and after adding C5a (*p* < 0.05). When PMX53 was added to the medium containing C5a, the axonal growth speed significantly decreased when compared to the control and remained stable between 24 and 48 h (Figure 8).

## 4. Discussion

This study shows that C5aR is expressed in neurons of hippocampal formation and that C5a enhances the injured axonal growth.

In situ hybridization combined with immunohistochemistry have already demonstrated that most neurons in the hippocampal formation constitutively express C5a receptors, which responded to C5a with increased calcium fluxes [22]. C5aR expression has also been reported in the spinal cord on motor neurons, and the microglia and its expression was significantly upregulated in the lumbar spinal cord following spinal cord injury [28]. Thus, C5aR expression in neurons is not a new finding. However, the novelty of this work is the demonstration that C5a phosphorylates its receptor on neurons and subsequently enhances the injured axonal growth. This investigation was possible thanks to the 3D-printed microfluidic device, which has been previously used as an in vitro model of axonal growth [25]. This model allowed us to (1) seed the cells in the upper chamber; (2) isolate and direct the axonal growth in microchannels of a separate compartment; (3) make a mechanical injury of the axons in the central channel and control their growth; and (4) apply a C5a in the lower chamber to create a gradient over 48 h between the upper and lower chambers through the microchannels. Use of this microfluidic device demonstrated that when C5a was applied in the lower chamber, the axons grew from the upper chamber towards C5a in the lower chamber through the microchannels. However, adding the C5a antagonist, PMX53, inhibited the C5aR phosphorylation in the cell culture and significantly inhibited the axonal growth. This highlights that the axonal growth is C5a-dependent. Surprisingly, although to a lesser extent than after adding C5a, adding the culture medium also enhanced the C5aR phosphorylation. This could be due to the secretion of C5a by astrocytes and neurons as reported previously [29,30]. Of note, these cells represent 7 and 93% of our cell cultures, respectively.

Our work demonstrates that C5a significantly enhanced the axonal growth after a mechanical injury. Indeed, while the average axonal growth speed of the control was 13 µm/h, adding C5a enhanced the axonal growth speed to 35 µm/h. This is of particular interest as adult central nervous system neurons have a weak axonal regeneration capacity with a reported speed ranging from ~2 to 10.7 µm/h [31,32]. Differences in the experimental designs used might explain this difference in the axonal growth speed between our results and previously reported data. Nevertheless, this highlights that C5a modulates the axonal growth after axotomy, which is of prime importance in case of injury to the central nervous system. This was investigated by injecting C5a intraperitoneally before or after spinal cord injury. When C5a was injected 24 h before or immediately after injury, the locomotor function, assessed for 9 weeks after SCI, was significantly impaired. However, when the treatment with C5a took place 24 h after injury, locomotor function improved significantly. This highlights that the delayed post-injury administration of C5a improves regeneration and functional recovery after spinal cord injury in mice [23].

C5a has been reported to have other effects on neurons, including a neuroprotective effect. Indeed, while adding C5a (50–100 nM) to cultured neurons significantly enhanced neurite outgrowth, it also inhibited caspase-3-mediated neuron apoptosis [23,33]. Similarly, pre-treatment of murine cortico-hippocampal neuronal cultures with C5a protected against glutamate-mediated apoptosis [34]. A confirmation of this effect was reported in C5aR knockout mice which confirmed that C5a deficiency renders C5aR knockout animals more susceptible to apoptotic injury in vivo [35]. Our findings are in line with these data as the observed effects in our study were obtained with the same C5a concentration (50 nM).

Notwithstanding the C5a-enhanced injured axonal speed in vitro, understanding C5a effects in vivo is much more difficult. This is due to the fact that, after spinal cord injury, C5a has been demonstrated to have time-dependent effects [18,23]. The initial effects have been ascribed to C5a implication in neuroprotection and inflammation [22,36,37] while the delayed effects are due to its implication in the improvement of regeneration and functional recovery after spinal cord injury [23]. This explains why some studies targeted C5a in the acute injury phase, as it may have deleterious effects and may hinder the delayed regeneration stage [16,17]. Pharmacological blockade or genetic ablation of the C5a receptor (C5aR) in mice significantly improved functional recovery [17]. These functional benefits corresponded with reduced inflammatory cytokine levels and monocyte-derived macrophage infiltration early after injury. Conversely, prolonged C5aR blockade for up to 21 days post-injury or permanent blockade in C5aR knock-out impaired the formation of the astrogliosis scar around the lesion core, exacerbated peripheral immune cell infiltration and lesion size, and reduced functional recovery [18].

It should be noted, however, that our results are obtained in vitro and the neurons used here are completely isolated/protected from the immune system/inflammatory events reported in vivo. Thus, our results cannot be compared to the clinical situation. However, this work highlights that inhibiting C5a, which provides neuroprotection and enhances axonal growth, may not be the best option in the therapeutic treatment of spinal cord injury. These results rather represent a future research direction.

In this context, the enhancement of axonal growth appears pivotal. The effect of C5a on axonal growth can be direct, as reported above, or indirect through inducing cellular release of neurotrophic factors. While our data demonstrate a direct effect of C5a by interacting with its receptor and enhancing axonal growth, the indirect effect has been illustrated in a previous study where C5a stimulated NGF secretion from dental pulp fibroblasts, leading to nerve sprouting and pulp nerve regeneration [24]. Similarly, astrocytes are known to secrete growth factors such as NGF and proliferate following C5a stimulation through STAT-3 pathway signalization [17,38]. Thus, activation of astrocytes by C5a could lead to release of growth factors by these cells and subsequently increase axonal growth. While the link between astrocyte activation by C5a, neurotrophic factor release, and axonal growth needs to be investigated, this highlights that C5a and its receptor also indirectly contribute to the outgrowth of peripheral axons [24].

Finally, while axonal regeneration is very limited in adult neurons as compared to the developing tissue, the cells obtained in this study are mainly mature neurons and the observed effects of C5a on the enhancement of axonal growth is of an added value for future investigations in vivo.

Thus, while C5a has the potential to stimulate axonal regeneration of mature neurons in vitro, and its delayed injection after spinal cord injury enhanced axonal growth and improved locomotor function, future studies are scheduled to investigate the effect of C5a sustained release from a specifically designed scaffold on healing and locomotor recovery after spinal cord injury.

## 5. Conclusions

Our findings highlight a new role of C5a in axonal regeneration by increasing the length and speed of axonal growth in vitro. These results may provide a valuable future therapeutic tool in CNS injury to repair neural networks over long distances and within the inhibitory environment of the adult CNS.

## Figures and Tables

**Table 1:** Primers used for gene expression by RT-PCR.

	Forward Primer	Reverse Primer
C5aR	5′—ACCATTCCGTCCTTCGT—3′	5′—CACCACTTTGAGCGTCTT—3′
GAPDH	5′—GCAAGTTCAACGGCACAG—3′	5′—CGCCAGTAGACTCCACGAC—3′

## Data Availability

Data are contained within the article.

## References

[1] Moreland T., Poulain F.E. (2022). To Stick or Not to Stick: The Multiple Roles of Cell Adhesion Molecules in Neural Circuit Assembly. Front. Neurosci..

[2] Yuasa-Kawada J., Kinoshita-Kawada M., Tsuboi Y., Wu J.Y. (2022). Neuronal Guidance Genes in Health and Diseases. Protein Cell.

[3] Bradke F. (2022). Mechanisms of Axon Growth and Regeneration: Moving between Development and Disease. J. Neurosci..

[4] Tedeschi A., Bradke F. (2017). Spatial and Temporal Arrangement of Neuronal Intrinsic and Extrinsic Mechanisms Controlling Axon Regeneration. Curr. Opin. Neurobiol..

[5] Alto L.T., Havton L.A., Conner J.M., Hollis E.R., Blesch A., Tuszynski M.H. (2009). Chemotropic Guidance Facilitates Axonal Regeneration and Synapse Formation after Spinal Cord Injury. Nat. Neurosci..

[6] Cafferty W.B.J., McGee A.W., Strittmatter S.M. (2008). Axonal Growth Therapeutics: Regeneration or Sprouting or Plasticity?. Trends Neurosci..

[7] Zhang Y., Al Mamun A., Yuan Y., Lu Q., Xiong J., Yang S., Wu C., Wu Y., Wang J. (2021). Acute Spinal Cord Injury: Pathophysiology and Pharmacological Intervention. Mol. Med. Rep..

[8] Shang Z., Wang M., Zhang B., Wang X., Wanyan P. (2022). Clinical Translation of Stem Cell Therapy for Spinal Cord Injury Still Premature: Results from a Single-Arm Meta-Analysis Based on 62 Clinical Trials. BMC Med..

[9] Zheng Y., Gallegos C.M., Xue H., Li S., Kim D.H., Zhou H., Xia X., Liu Y., Cao Q. (2022). Transplantation of Human Induced Pluripotent Stem Cell-Derived Neural Progenitor Cells Promotes Forelimb Functional Recovery after Cervical Spinal Cord Injury. Cells.

[10] Haggerty A.E., Oudega M. (2013). Biomaterials for Spinal Cord Repair. Neurosci. Bull..

[11] Ziemba A.M., Gilbert R.J. (2017). Biomaterials for Local, Controlled Drug Delivery to the Injured Spinal Cord. Front. Pharmacol..

[12] Bergmann M., Jeanneau C., Giraud T., Richard G., About I. (2020). Complement Activation Links Inflammation to Dental Tissue Regeneration. Clin. Oral. Investig..

[13] Guo R.-F., Ward P.A. (2005). Role of C5a in Inflammatory Responses. Annu. Rev. Immunol..

[14] Le Fournis C., Jeanneau C., Roumani S., Giraud T., About I. (2020). Pulp Fibroblast Contribution to the Local Control of Pulp Inflammation via Complement Activation. J. Endod..

[15] Le Fournis C., Hadjichristou C., Jeanneau C., About I. (2019). Human Pulp Fibroblast Implication in Phagocytosis via Complement Activation. J. Endod..

[16] Biggins P.J.C., Brennan F.H., Taylor S.M., Woodruff T.M., Ruitenberg M.J. (2017). The Alternative Receptor for Complement Component 5a, C5aR2, Conveys Neuroprotection in Traumatic Spinal Cord Injury. J. Neurotrauma.

[17] Brennan F.H., Gordon R., Lao H.W., Biggins P.J., Taylor S.M., Franklin R.J.M., Woodruff T.M., Ruitenberg M.J. (2015). The Complement Receptor C5aR Controls Acute Inflammation and Astrogliosis Following Spinal Cord Injury. J. Neurosci..

[18] Ulndreaj A., Marbourg J.M., Vidal P.M. (2015). The Complement Receptor C5aR Has a Dual, Time-Dependent Effect on the Outcome of Spinal Cord Injury. J. Neurosci..

[19] Coulthard L.G., Hawksworth O.A., Li R., Balachandran A., Lee J.D., Sepehrband F., Kurniawan N., Jeanes A., Simmons D.G., Wolvetang E. (2017). Complement C5aR1 Signaling Promotes Polarization and Proliferation of Embryonic Neural Progenitor Cells through PKCζ. J. Neurosci..

[20] Nataf S., Levison S., Barnum S. (2001). Expression of the Anaphylatoxin C5a Receptor in the Oligodendrocyte Lineage. Brain Res..

[21] Crane J., Baiquni G., Sullivan R., Lee J., Sah P., Taylor S., Noakes P., Woodruff T. (2009). The C5a Anaphylatoxin Receptor CD88 Is Expressed in Presynaptic Terminals of Hippocampal Mossy Fibres. J. Neuroinflamm..

[22] O’Barr S.A., Caguioa J., Gruol D., Perkins G., Ember J.A., Hugli T., Cooper N.R. (2001). Neuronal Expression of a Functional Receptor for the C5a Complement Activation Fragment. J. Immunol..

[23] Guo Q., Cheng J., Zhang J., Su B., Bian C., Lin S., Zhong C. (2013). Delayed Post-Injury Administration of C5a Improves Regeneration and Functional Recovery after Spinal Cord Injury in Mice. Clin. Exp. Immunol..

[24] Chmilewsky F., Ayaz W., Appiah J., About I., Chung S. (2016). Nerve Growth Factor Secretion From Pulp Fibroblasts Is Modulated by Complement C5a Receptor and Implied in Neurite Outgrowth. Sci. Rep..

[25] Taylor A.M., Blurton-Jones M., Rhee S.W., Cribbs D.H., Cotman C.W., Jeon N.L. (2005). A Microfluidic Culture Platform for CNS Axonal Injury, Regeneration and Transport. Nat. Methods.

[26] Virlogeux A., Moutaux E., Christaller W., Genoux A., Bruyère J., Fino E., Charlot B., Cazorla M., Saudou F. (2018). Reconstituting Corticostriatal Network On-a-Chip Reveals the Contribution of the Presynaptic Compartment to Huntington’s Disease. Cell Rep..

[27] Meijering E., Jacob M., Sarria J.-C.F., Steiner P., Hirling H., Unser M. (2004). Design and Validation of a Tool for Neurite Tracing and Analysis in Fluorescence Microscopy Images. Cytometry A.

[28] Dong Q., Sun L., Peng L., Yan B., Lv J., Wang G., Gong S. (2015). Expression of C5a and Its Receptor Following Spinal Cord Ischemia Reperfusion Injury in the Rat. Spinal. Cord..

[29] Thomas A., Gasque P., Vaudry D., Gonzalez B., Fontaine M. (2000). Expression of a Complete and Functional Complement System by Human Neuronal Cells in Vitro. Int. Immunol..

[30] Yu J.X., Bradt B.M., Cooper N.R. (2002). Constitutive Expression of Proinflammatory Complement Components by Subsets of Neurons in the Central Nervous System. J. Neuroimmunol..

[31] Li D., Zhang S., Yao Y., Xiang Y., Ma X., Wei X., Yan H., Liu X. (2017). Sigma-1 Receptor Agonist Increases Axon Outgrowth of Hippocampal Neurons via Voltage-gated Calcium Ions Channels. CNS Neurosci. Ther..

[32] Nieuwenhuis B., Barber A.C., Evans R.S., Pearson C.S., Fuchs J., MacQueen A.R., van Erp S., Haenzi B., Hulshof L.A., Osborne A. (2020). PI 3-kinase Delta Enhances Axonal PIP 3 to Support Axon Regeneration in the Adult CNS. EMBO Mol. Med..

[33] Mukherjee P., Pasinetti G.M. (2001). Complement Anaphylatoxin C5a Neuroprotects through Mitogen-Activated Protein Kinase-Dependent Inhibition of Caspase 3. J. Neurochem..

[34] Tocco G., Musleh W., Sakhi S., Schreiber S.S., Baudry M., Pasinetti G.M. (1997). Complement and Glutamate Neurotoxicity. Genotypic Influences of C5 in a Mouse Model of Hippocampal Neurodegeneration. Mol. Chem. Neuropathol..

[35] Mukherjee P., Thomas S., Pasinetti G.M. (2008). Complement Anaphylatoxin C5a Neuroprotects through Regulation of Glutamate Receptor Subunit 2 in Vitro and in Vivo. J. Neuroinflamm..

[36] Bamberg C.E., Mackay C.R., Lee H., Zahra D., Jackson J., Lim Y.S., Whitfeld P.L., Craig S., Corsini E., Lu B. (2010). The C5a Receptor (C5aR) C5L2 Is a Modulator of C5aR-Mediated Signal Transduction. J. Biol. Chem..

[37] Donnelly D.J., Popovich P.G. (2008). Inflammation and Its Role in Neuroprotection, Axonal Regeneration and Functional Recovery after Spinal Cord Injury. Exp. Neurol..

[38] Jauneau A., Ischenko A., Chatagner A., Benard M., Chan P., Schouft M., Patte C., Vaudry H., Fontaine M. (2006). Interleukin-1beta and Anaphylatoxins Exert a Synergistic Effect on NGF Expression by Astrocytes. J. Neuroinflamm..

